# Two new species of *Micrencaustes* Crotch, subgenus *Mimencaustes* Heller from China (Coleoptera, Erotylidae, Encaustini)

**DOI:** 10.3897/zookeys.391.7025

**Published:** 2014-03-20

**Authors:** Zhao-Na Meng, Guo-Dong Ren, Jing Li

**Affiliations:** 1 College of Plant Protection, post-doctoral research center of Crop Science, Agricultural University of Hebei, Baoding, Hebei, 071002, P. R. China; 2 College of Life Sciences, Hebei University, Baoding, Hebei 071002, P. R. China

**Keywords:** Coleoptera, Erotylidae, *Micrencaustes*, *Mimencaustes*, identification key, new species, China

## Abstract

Two new species *Micrencaustes (Mimencaustes) renshiae* **sp. n.** and *Micrencaustes (Mimencaustes) biomaculata* **sp. n.** are described and illustrated from China. A key to Chinese species of subgenus *Mimencaustes* Heller is provided.

## Introduction

To date, only 2 subgenera of the genus *Micrencaustes* Crotch, 1876 are known (Crotch 1876; Heller 1918). One of the two subgenera, *Mimencaustes* was erected by Heller for *Micrencaustes dehaanii* (Castelnau, 1840), as the type species. It differs from *Micrencaustes* (s. str.) by the presence of mesocoxal lines. The subgenus *Mimencaustes* included 8 species worldwide (Castelnau 1840; Gorham 1883; Heller 1914; Heller 1918; Araki 1941; Chûjô and Chûjô 1989; Osawa and Chûjô 1990; Li and Ren 2006). They are mainly distributed in Asia. But *Micrencaustes (Mimencaustes) torquata* Gorham 1883 was originally stated to inhabit West Africa. Arrow (1925) pointed out this was probably a mistake in labeling the type specimen. The presence of this genus in West Africa is uncertain. Previously, only two species, *Micrencaustes (Mimencaustes) taiwana* Araki 1941 and *Micrencaustes (Mimencaustes) acridentata* Li & Ren, 2006, are known from China.

In this work, two new species of the subgenus *Mimencaustes* are described and illustrated: *Micrencaustes (Mimencaustes) renshiae* sp. n. from Hainan Province and *Micrencaustes (Mimencaustes) biomaculata* sp. n. from Yunnan Province, China. Two species, *Micrencaustes (Mimencaustes) dehaanii* (Castelnau, 1840) and *Micrencaustes (Mimencaustes) wunderlichi* Heller, 1918 are recorded from China for the first time.

## Material and methods

The specimens examined in this paper were collected in a wide variety of woodland fungus, in crevices under bark or in other retreats by splitting and sifting. They were killed with ethyl acetate and dried. For an examination of the male or female genitalia, the abdominal segments were detached from the body after softening in hot water. Morphological figures were prepared using a Nikon SMZ1500 stereomicroscope; habitus photos were taken with a Nikon D7000 camera. All measurements are given in millimeters. The specimens treated in this study are deposited in the Museum of Hebei University (MHU), Hebei, P. R. China.

Morphological terminology predominantly follows Wegrzynowicz (1997) with changes according to Skelley and Leschen (2007).

The measurements of proportions are abbreviated as follows:

pl/pw – pronotum length/width ratio.

## Taxonomy

### Key to Chinese species of the subgenus *Mimencaustes*

**Table:** 

1	Body entirely dark, without mark	*Micrencaustes (Mimencaustes) dehaanii* (New record to China)
–	Body with marks	2
2	The marks on pronotum and elytron	*Micrencaustes (Mimencaustes) taiwana*
–	The marks only on pronotum or elytron	3
3	Pronotum with marks	4
–	Elytron with marks	5
4	Head without orange mark, prosternal femoral lines surpassing the front edge of coxae	*Micrencaustes (Mimencaustes) acridentata*
–	Head with orange mark, prosternal femoral lines reaching the front edge of coxae	*Micrencaustes (Mimencaustes) renshiae* sp. n.
5	Basal mark of elytron smaller, without black spots near anterior border	*Micrencaustes (Mimencaustes) wunderlichi* (New record to China)
–	Basal mark of elytron bigger, with 2 black spots near anterior border	*Micrencaustes (Mimencaustes) biomaculata* sp. n.

#### Micrencaustes (Mimencaustes) renshiae sp. n.

http://zoobank.org/F3871C6A-063C-493E-B7EE-259047C1D12A

http://species-id.net/wiki/Micrencaustes_renshiae

##### Type material.

Holotype. male, CHINA: Hainan Province, Baisha County, 19.2248°N, 109.4514°E, alt. 450 m, 27 May 2008, Yi-Bin Ba & Jun-Tong Lang leg. (MHU). Paratypes. 3 males, 4 females, same data as holotype (MHU).

##### Description.

**Figures 1.**
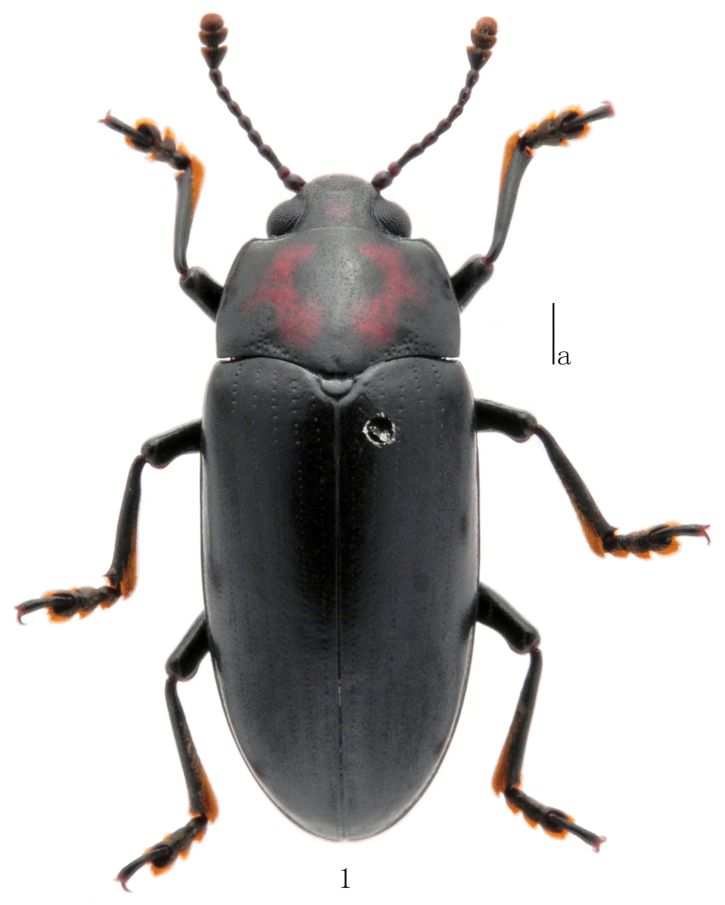
Habitus of *Micrencaustes (Mimencaustes) renshiae* Scale: a = 1 mm.

Body (Fig. 1) moderately elongate, length: 10.5–12.4 mm, width: 4.1–4.5 mm; widest at base of elytra, general color dark, slightly shining. Head with an irregular orange mark between eyes. Pronotum with two longitudinal, curved orange marks, each bearing a short branch in the middle.

**Figures 2–11.**
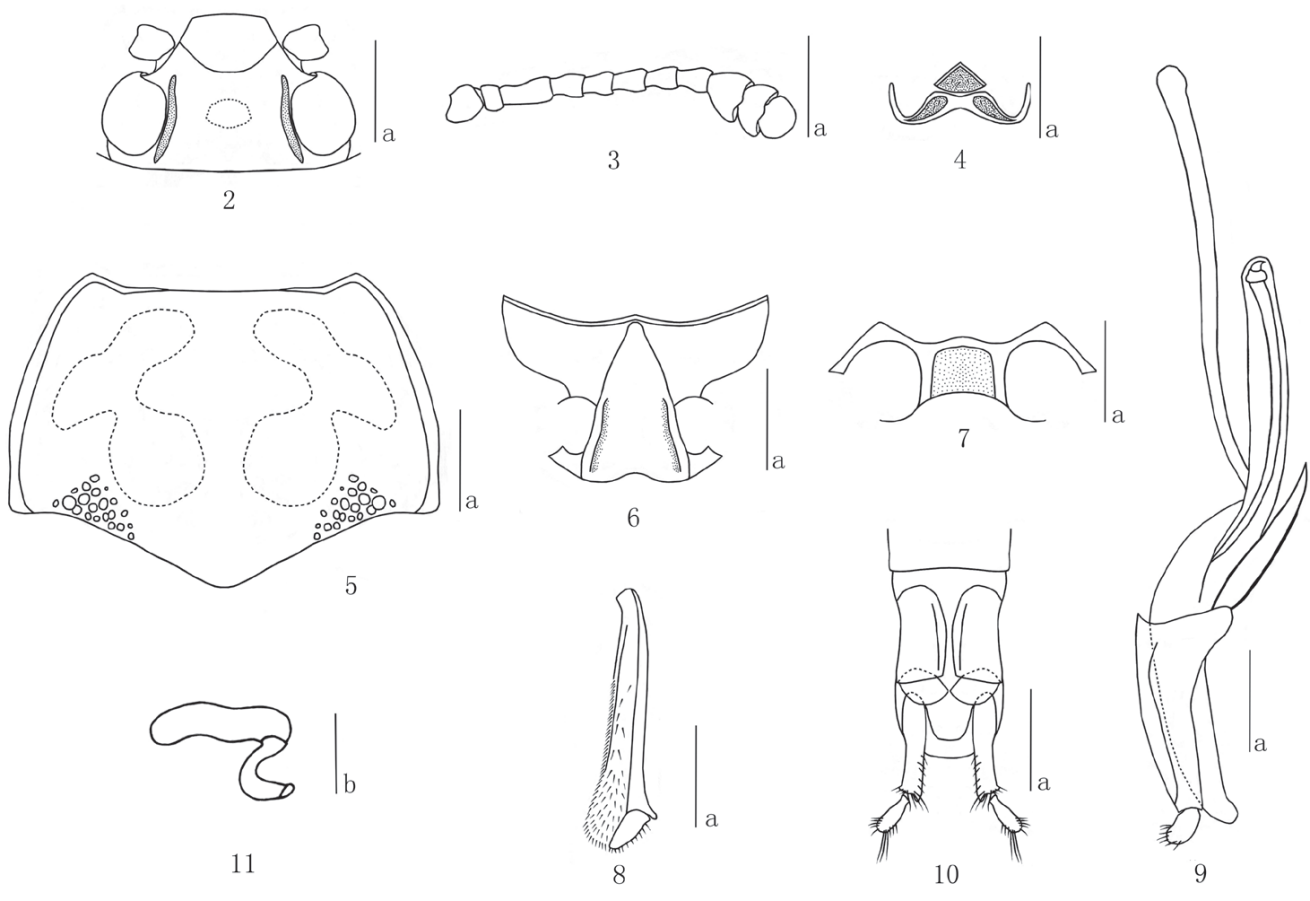
*Micrencaustes (Mimencaustes) renshiae* sp.n. **2** head **3** antenna **4** mentum **5** pronotum **6** prosternum **7** mesoventrite **8** mesotibia **9** aedeagus in lateral views **10** female genitalia in ventral view **11** female spermatheca Scale bars: **a** = 1 mm, **b** = 0.5 mm.

Head (Fig. 2) strongly and sparsely punctured on vertex, with ocular lines. Clypeus strongly and rather closely punctured, with anterior border feebly emarginate, with a fovea on each side of the base. Eyes large, moderately prominent laterally. Antennae (Fig. 3) extending posterior border of pronotum; antennomere III about 1.75 times as long as IV; antennomere IX asymmetrical, almost triangular; antennomere X bowl-shaped; antennomere XI hemispherical, narrower than antennomere X, slightly constricted in middle; relative lengths of antennomeres II–XI: 12: 35: 20: 19: 19: 19: 20: 18: 16: 19. Maxillary palp terminal segment triangular, sides rounded, nearly 2.7 times as wide as long. Mentum (Fig. 4) with plate triangular, sides concave, with coarse punctures and setae; submentum roughly punctured, with long golden setae.

Pronotum (Fig. 5) widest at base (pl/pw = 0.69–0.74); lateral margin slightly curved; pronotal anterior margin straight in the middle; basal margin weakly sinuate. Pronotum finely punctate, punctures evenly scattered; with a group of coarse punctures on each side of base area. Anterior angles projected; posterior angles nearly rectangular.

Prosternum (Fig. 6) coarsely and sparsely punctured on lateral areas, with some oblique rugae; an irregular depression in the middle of base area; surface with golden pubescence. Prosternal process triangular, strongly emarginated at apical border, produced into a blunt point at the base. Prosternal femoral lines almost straight, converging anteriorly and reaching the front edge of coxae.

Scutellum pentagonal, with fine and spare punctures.

Elytra widest near base, then gradually narrowing to apex. Each elytron with 8 striae; strial punctures stronger at basal part, gradually weakened apically and disappeared before extremity; intervals finely punctured and wrinkled.

Mesoventrite (Fig. 7) broad, with a median quadrate depression; mesocoxal lines short; sternum with fine and sparse punctures.

Mesotibia (Fig. 8) with outer edge of apex acutely toothed.

Male genitalia (Fig. 9) with median lobe weakly curved; narrowed to a point in lateral view; median strut about 1.31 times as long as median lobe.

Female genitalia (Fig. 10) with narrow styli at apex of coxite, and styli rounded apically, covered with setae at apex. Female spermatheca as in Fig. 11.

##### Distribution.

Known only from the type locality (China: Hainan Province, Baisha County).

##### Diagnosis.

*Micrencaustes (Mimencaustes) renshiae* is most similar to *Micrencaustes (Mimencaustes) acridentata* Li & Ren, 2006, due to the similar form and color pattern of pronotum. The new species can be distinguished from it by the head with orange mark; prosternum with an irregular depression in the middle of base area; prosternal femoral lines reaching the front edge of coxae. *Micrencaustes (Mimencaustes) acridentata* without orange mark on head; prosternum with a distinctly depressed in the middle; prosternal femoral lines surpassing the front edge of coxae.

##### Etymology.

This species is named in honor of Mr. Guo-Dong Ren, teacher of author Jing Li, who helped a lot during our work.

#### Micrencaustes (Mimencaustes) biomaculata sp. n.

http://zoobank.org/B973810F-7181-4236-9605-ABFFC6E5B3B1

http://species-id.net/wiki/Micrencaustes_biomaculata

##### Type material.

Holotype. male, CHINA: Yunnan Province, Eryuan County, 26.1111°N, 99.9510°E, alt. 1870 m, 17 August 2008, Ji-Shan Xu leg. (MHU). Paratypes. 3 males, 6 females, same data as holotype (MHU).

##### Description.

**Figures 12.**
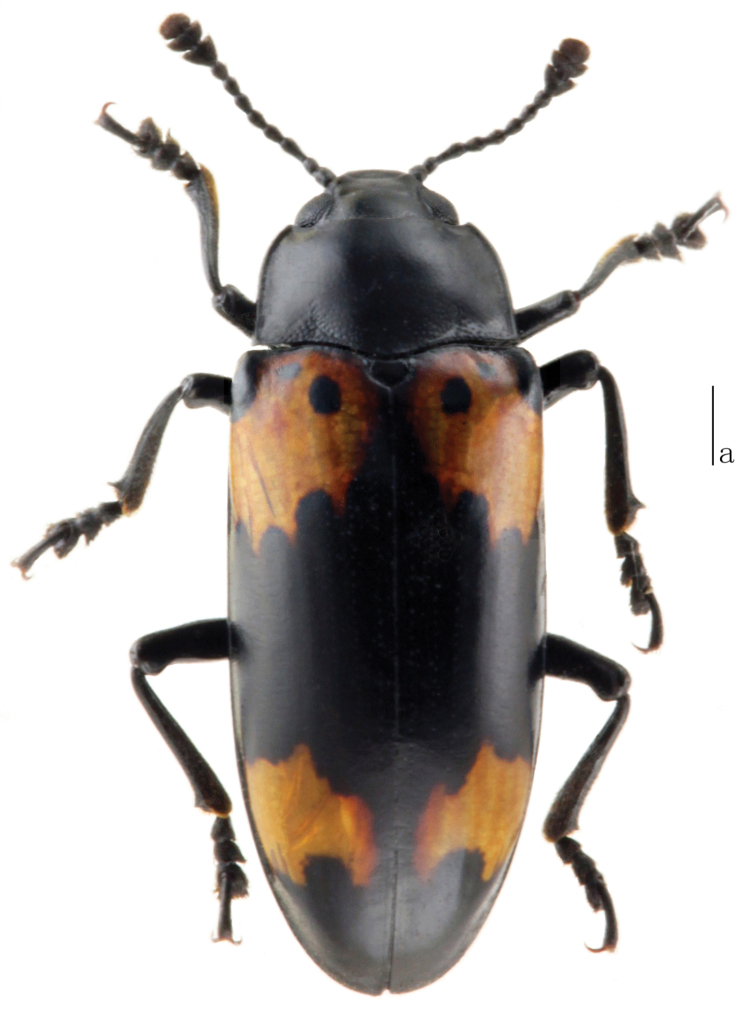
Habitus of *Micrencaustes (Mimencaustes) biomaculata* Scale: a = 1 mm.

Body (Fig. 12) oblong oval, moderately convex, length: 10.8–12.5 mm, width: 3.5–4.4 mm; general color dark, moderately shining. Each elytron with 2 orange bands; anterior band reaching lateral and basal margins, leaving a black part at humerus, with 2 black spots near anterior border, with 4 teeth at posterior border; posterior band at four fifths length of elytron, extending from the striae I to near the lateral border, with 2 teeth at anterior border, with 3 teeth at posterior border.

**Figures 13–22.**
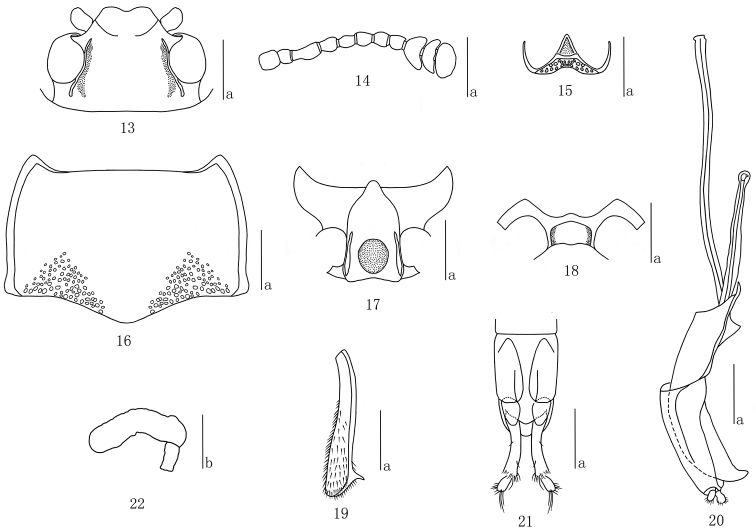
*Micrencaustes (Mimencaustes) biomaculata* sp. n. **13** head **14** antenna **15** mentum **16** pronotum **17** prosternum **18** mesoventrite **19** mesotibia **20** aedeagus in lateral views **21** female genitalia in ventral view **22** female spermatheca Scale bars: **a** = 1 mm, **b** = 0.5 mm.

Head (Fig. 13) sparsely punctured on vertex, closely at base, with ocular lines. Clypeus strongly and closely punctured, with anterior border feebly emarginate, with a fovea on each side of the base. Eyes large, rather prominent laterally. Antennae (Fig. 14) reaching basal 0.25 of pronotum; antennomere III about 1.69 times as long as IV; antennomere IX asymmetrical, almost triangular; antennomere X crescent-shaped; antennomere XI hemispherical, narrower than antennomere X, slightly constricted in middle; relative lengths of antennomeres II–XI: 12: 27: 16: 16: 17: 16: 14: 18: 13: 20. Maxillary palp terminal segment broadly triangular, nearly 3.2 times as wide as long. Mentum (Fig. 15) with plate triangular, sides concave, with rather coarse punctures and setae; submentum roughly punctured, with short golden setae.

Pronotum (Fig. 16) widest at middle (pl/pw = 0.67–0.72); sides almost parallel on posterior half and narrowing toward apex, strongly margined; anterior margin straight in the middle, margined behind eyes; basal margin weakly sinuate. Pronotum finely and sparsely punctuated, with a group of coarse punctures on each side of base area, with punctate longitudinal median areas. Anterior angles projected; posterior angles nearly rectangular.

Prosternum (Fig. 17) coarsely and sparsely punctured on lateral areas, with some oblique rugae; an irregular depression in the middle of base area; surface with golden pubescence. Prosternal process triangular, strongly emarginated at apical border, produced into a blunt point at the base. Prosternal femoral lines converging anteriorly and reaching the front edge of coxae.

Scutellum nearly pentagonal, sparely punctured.

Elytra widest at base, sides almost parallel at two thirds from base, then gradually narrowing to apex; strial punctures fine at basal part and disappeared before extremity; intervals finely punctured and distinctly wrinkled.

Mesoventrite (Fig. 18) broad, with a median pentagonal depression; mesocoxal lines moderate length; sparsely punctured.

Mesotibia (Fig. 19) with outer edge of apex acutely toothed.

Male genitalia (Fig. 20) with median lobe curved; narrowed to a hook at apex in lateral view; median strut about 1.43 times as long as median lobe.

Female genitalia (Fig. 21) with narrow styli at apex of coxite, and styli rounded apically, covered with setae at apex. Female spermatheca as in Fig. 22.

##### Distribution.

Known only from the type locality (China: Yunnan Province, Eryuan County).

##### Diagnosis.

The new species is most similar to *Micrencaustes (Mimencaustes) michioi* Osawa & Chûjô, 1990, but can be distinguished from it by body moderately shining; pronotum with anterior margin margined behind eyes, without impunctate longitudinal median areas; mesotibia with outer edge of apex acutely toothed. In *Micrencaustes (Mimencaustes) michioi*, the body fairly shining; pronotum with anterior margin immarginate, with impunctate longitudinal median areas; every tibia with outer edge of apex acutely toothed.

##### Etymology.

The species is named with 2 black spots near anterior border of basal mark of elytron.

#### Micrencaustes (Mimencaustes) dehaanii

(Castelnau, 1840) (New record to China)

http://species-id.net/wiki/Micrencaustes_dehaanii

Engis dehaanii Castelnau, 1840: 15.
Encaustes dehaanii , Lacordaire 1842: 42.
Micrencaustes dehaanii , Crotch 1876: 572.
Micrencaustes (Mimencaustes) dehaanii , Heller 1918 (1920): 10.

##### Material examined.

1 female, CHINA: Yunnan Province, Lianghe County, 24.8070°N, 98.2949°E, 3 May 1957, Bong Faye Love leg.

##### Distribution.

China (Yunnan), Java, Malaysia, Thailand, Singapore, Vietnam, Laos, India, Sikkim.

#### Micrencaustes (Mimencaustes) wunderlichi

Heller, 1918 (New record to China)

http://species-id.net/wiki/Micrencaustes_wunderlichi

Micrencaustes (Mimencaustes) wunderlichi Heller, 1918 (1920): 23.
Micrencaustes wunderlichi , Arrow 1926: 254.

##### Material examined.

1 male, CHINA: Yunnan Province, Lushui County, 25.9667°N, 98.8167°E, 11 May 2004, Zi-Zhong Yang leg (MHU).

##### Distribution.

China (Yunnan), Java, Indonesia.

## Supplementary Material

XML Treatment for Micrencaustes (Mimencaustes) renshiae

XML Treatment for Micrencaustes (Mimencaustes) biomaculata

XML Treatment for Micrencaustes (Mimencaustes) dehaanii

XML Treatment for Micrencaustes (Mimencaustes) wunderlichi
